# EFFECT OF STIGMA ON OUTCOMES OF A MEMORY TRAINING INTERVENTION

**DOI:** 10.1093/geroni/igz038.2438

**Published:** 2019-11-08

**Authors:** Graham J McDougall, Ian M McDonough, Kyle Kraemer

**Affiliations:** 1 Florida State University, Tallahassee, Florida, United States; 2 University of Alabama, Tuscaloosa, Alabama, United States

## Abstract

Cultural stereotypes equated with aging that emphasize decreasing competence and increasing forgetfulness can be threatening to older adults. Even brief exposure via entertainment media or the patronizing behavior of others may induce stigma in elders and thereby impair memory and executive functions. The sample was recruited for an RCT known as Senior WISE and conducted in Central Texas. The average age was 75 years and average of 14 years of education. Sex and minority status were consistent across groups. Data were analyzed using SPSS, v21. First, Pearson Rs were calculated between stigma (MIA Anxiety subscale) and memory outcomes at baseline. Stigma related significantly to Rivermead (RBMT), HVLT, and Memory Self-Efficacy (MSQ). Then, after controlling for the effects of trait anxiety, stigma explained a significant portion of the variance within scores on the RBMT (β = -.139, R2 MIA Change = .016, p = .037), HVLT (β = -.145 R2 Change = .017, p =.032), and MSQ-35 (β = -.253, R2 Change = .053, p < .001). Change in stigma was significantly associated with change in HVLT scores among those in the memory training group, r(105) = -.228, p = .018. Reductions in stigma were related to increases in HVLT score, β = -.14, F(1, 201) = 3.021, p = .084, R2 Change = .015; however, the overall regression model was not a good predictor of HVLT change, F(4, 201) = 1.793, p = .132, R2 = .034. Stigma is a high priority area of scientific inquiry and a self-fulfilling prophecy.

